# Rice leaf endophytic *Microbacterium testaceum*: Antifungal actinobacterium confers immunocompetence against rice blast disease

**DOI:** 10.3389/fmicb.2022.1035602

**Published:** 2022-12-21

**Authors:** Asharani Patel, Kuleshwar Prasad Sahu, Sahil Mehta, Alexander Balamurugan, Mukesh Kumar, Neelam Sheoran, Shanu Kumar, Charishma Krishnappa, Mushineni Ashajyothi, Aditi Kundu, Tushar Goyal, Prabhakaran Narayanasamy, Aundy Kumar

**Affiliations:** ^1^ICAR-Indian Agricultural Research Institute, New Delhi, India; ^2^T-Stanes & Company Ltd., Coimbatore, India

**Keywords:** rice, endophytic *Microbacterium*, defense genes, volatiles, blast

## Abstract

Genetic and functional characteristics of rice leaf endophytic actinobacterial member, *Microbacterium* are described. Morphotyping, multilocus sequence analysis and transmission electron microscopy indicated the species identity of the endophytic bacterium, OsEnb-ALM-D18, as *Microbacterium testaceum*. The endophytic *Microbacterium* showed probiotic solubilization of plant nutrients/minerals, produced hydrolytic enzyme/phytohormones, and showed endophytism in rice seedlings. Further, the endophytic colonization by *M. testaceum* OsEnb-ALM-D18 was confirmed using reporter gene coding for green fluorescence protein. *Microbacterium* OsEnb-ALM-D18 showed volatilome-mediated antibiosis (95.5% mycelial inhibition) on *Magnaporthe oryzae*. Chemical profiling of *M. testaceum* OsEnb-ALM-D18 volatilome revealed the abundance of 9-Octadecenoic acid, Hexadecanoic acid, 4-Methyl-2-pentanol, and 2,5-Dihydro-thiophene. Upon endobacterization of rice seedlings, *M. testaceum* altered shoot and root phenotype suggestive of activated defense. Over 80.0% blast disease severity reduction was observed on the susceptible rice cultivar Pusa Basmati-1 upon foliar spray with *M. testaceum*. qPCR-based gene expression analysis showed induction of *Os*CERK1, *Os*PAD4, *Os*NPR1.3, and *Os*FMO1 suggestive of endophytic immunocompetence against blast disease. Moreover, *M. testaceum* OsEnb-ALM-D18 conferred immunocompetence, and antifungal antibiosis can be the future integrated blast management strategy.

## Graphical Abstract

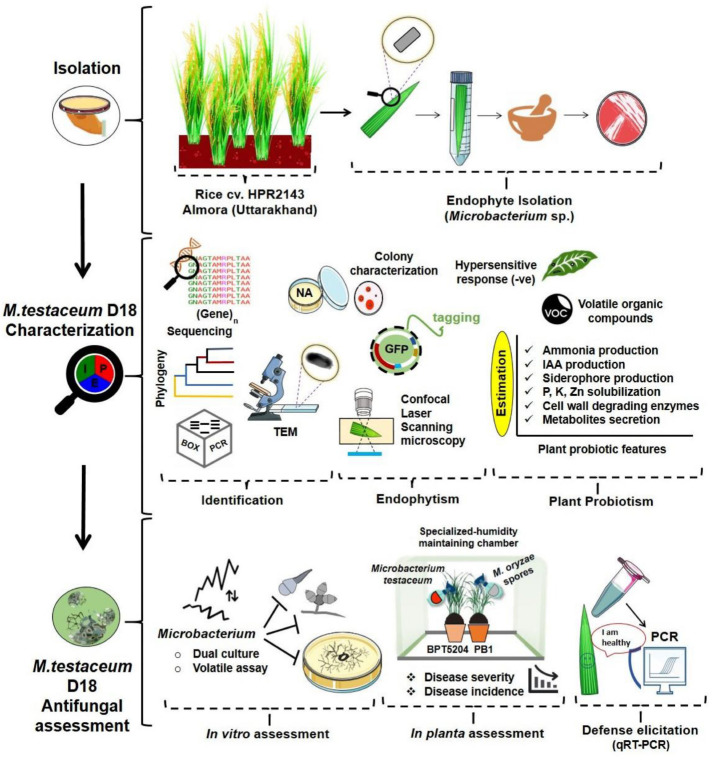

## Introduction

Rice (*Oryza sativa* L.; Family Poaceae) is the cereal staple that serves as the primary source of sustenance for more than half of the world’s population (Shah et al., 2021). Rice cultivation is constrained by several biotic and abiotic stresses causing yield and economic losses worldwide (Fahad et al., 2019; Savary et al., 2019). Among the biotic stresses, diseases incited by phytopathogenic fungi and bacteria have resulted in a significant production loss impacting the global food supply chain (Miah et al., 2013; Godfray et al., 2016; Jiehua et al., 2019; Neupane and Bhusal, 2021). Notably, rice blast disease incited by the hemibiotrophic ascomycete *Magnaporthe* is responsible for pre-harvest losses of about 10–30% (Rossman et al., 1990; Howard and Valent, 1996; Shan et al., 2013; Kirtphaiboon et al., 2021). The disease symptoms are elliptical-necrotic lesions on the leaf with diamond-shaped gray centers and a reddish-brown margin which is noticed on the vegetative leaf and reproductive panicle (Asibi et al., 2019). Traditionally, the blast mitigation strategies depend on the deployment of host resistance and the application of fungicide (Mehta et al., 2021). However, both approaches are inadequate to sustain rice production. The use of fungicides led to the persistence of harmful residues on grain, which prompted several nations to reject shipments of rice and resulted in financial losses for both traders and farmers (Zhang, 2007). The host resistance is not durable and often ineffective in many regions owing to the high genetic and pathogenic diversity of *Magnaporthe* (Hubballi et al., 2022). As a result, developing effective and sustainable strategies to combat blast disease assumes significance. In recent years, there is a demand for eco-friendly biocontrol methods for rice blast disease management (Skamnioti and Gurr, 2009; Chaiharn et al., 2020; Sahu et al., 2021a).

Biological control system exploiting naturally occurring endophytic microbes is one of the choices due to their innate ability to interact with the susceptible host niches and the suppression of pathogenic agents by direct antibiosis and indirect defense activation (Liu et al., 2007; Mano and Morisaki, 2008). In addition to probiotic effects on host plants, several endophytic bacteria have been reported to induce systemic response against pathogens in many dicots and monocot crops including rice (Sheoran et al., 2015, 2016; Agisha et al., 2017; Hirakue and Sugiyama, 2018; Li et al., 2019; Sarkar et al., 2019; Ashajyothi et al., 2020; Matsumoto et al., 2021; Patel et al., 2021). Recently, we published the antagonistic and host defense elicitation potential of rice-endophytic flavobacterial isolate *Chryseobacterium endophyticum* against rice blast (Kumar et al., 2021a). It is known that plant ecological functions including its defense against the pathogen are carried out by the synergistic effects of the plant microbiome, there are ample chances that other rice endophytic bacteria can have blasticidal potential. Considering this point, we focused on isolating other endophytes from rice foliage and evaluated their antifungal potential against *Magnaporthe oryzae*.

Among the rice endophytic bacterial communities, the genus *Microbacterium* belonging to the class, Actinobacteria is characterized by small, irregular, short, slender, rod-shaped Gram-positive bacterium (Komagata et al., 1964; Yamada and Komagata, 1972; Collins et al., 1983; Takeuchi and Hatano, 1998). In the past, a series of research publications have highlighted the *Microbacterium* as a promising microbial inoculant in agriculture that displays biostimulant activity on crops and antibiosis against pathogens. *Microbacterium* is also prolific for the production of volatile organic compounds that contributed to growth promotion and development (Barnett et al., 2006; Cordovez et al., 2018). Moreover, *Microbacterium* sp. isolated from rice as well as other plants has been used as an effective biocontrol agent against various plant pathogens by the production of a pectinolytic enzyme, antimicrobial substances, insoluble and soluble exopolysaccharides or degradation of toxic substances (Ueno et al., 1983; Matsuyama et al., 1999; Dikin et al., 2007; Cottyn et al., 2009; Silva et al., 2012; Walitang et al., 2017). The interaction of three different taxonomic groups of bacteria such as *Pantoea*, *Exiguobacterium*, and *Microbacterium* could reduce the disease severity caused by *Rhizoctonia solani* AG-8 (Barnett et al., 2006). Along with *Bacillus amyloliquefaciens*, *Microbacterium* reduced *Fusarium verticillioides* and the mycotoxin fumonisin B1 and B2 in maize grains (Sartori et al., 2012). Before the present study, leaf epiphytic *Microbacterium testaceum* was reported to elicit defense responses leading to blast disease suppression upon phyllo bacterization of rice cultivar Pusa Basmati-1 (Sahu et al., 2021b). However, published literature on the biocontrol potential of *M. testaceum* against blast disease is scanty. Therefore, an in-depth genetic and functional characterization of rice endophytic *M. testaceum* was carried out to develop a biostimulant for sustainable rice production. We further attempted to elucidate the mode of action of *M. testaceum* so that it can be incorporated into eco-friendly rice blast disease management options in the future.

## Materials and methods

### Bacterial strain

The bacterial strain OsEnb-ALM-D18 used in the present study was isolated from the leaf endosphere of rice (*O. sativa* L.) cultivar HPR2143 grown at Almora (Uttarakhand) as a natural endophyte (Kumar, 2021). 16S rRNA gene sequence was used for identification as *M. testaceum* and the sequence was submitted to NCBI GenBank with accession number MN889362.^1^ For routine lab work, culture was preserved at −80°C as glycerol stock was retrieved by streaking on plates of nutrient agar medium [NA, gL^–1^ Beef extract 3.0; Peptone 5.0; NaCl 5.0; Agar 15.0; pH 7.0 ± 0.2] and incubated at 28°C for 48 h.

### Confirmation of the identity of endophytic bacterium

#### Transmission electron microscopy

Using the negative-staining method (Tranum-Jensen, 1988), the bacterium *M. testaceum* OsEnb-ALM-D18 (hereinafter *M. testaceum* D18) was visualized and imaged in the transmission electron microscope (TEM) at magnifications of more than 60K X (Joel, Tokyo, Japan).

#### Genomic characterization by sequence analysis of multiple genes

##### Genomic DNA isolation

Briefly, *M. testaceum* D18 was grown in nutrient broth with constant agitation (100 rpm). Bacterial culture at mid-log phase (1.5 mL; 1.0 OD_*A600 nm*_) was centrifuged at 12,000 rpm for 3.0 min. Pelleted bacterial cells were washed with sterile saline and used for extracting genomic DNA. CTAB-method with slight modification was used to isolate bacterial genomic DNA (Sheoran et al., 2015; Munjal et al., 2016; Eke et al., 2019; Sahu et al., 2021b). Before PCR amplification, the integrity, purity, and yield of genomic DNA were tested spectrophotometrically (Biophotometer, Eppendorf, Hamburg, Germany) and electrophoretically. Finally, a 100 ng μL^–1^ concentration of the genomic DNA was prepared and used as an amplification template.

##### Multigene sequencing and identification

A total of eight *Microbacterium*-specific housekeeping genes were taken for this study. The primers were designed using Primer3plus, and the same were synthesized and used.^2^ Supplementary Table 1 lists the oligo primers that were used in the PCR amplification. PCR reaction mixture contains-Promega PCR buffer 1X; MgCl_2_ 1.5 mM; dNTPs 200 mM; Taq polymerase 1.0 U; forward and reverse primers 10-pmol each; genomic DNA 100 ng; and mQ water to bring the total volume to 50 μL. The following temperature conditions parameters were used for PCR amplification in a thermal cycler (MasterCycler ProS, Eppendorf, Hamburg, Germany): initial denaturation at 95°C for 5 min, followed by 35 cycles of 94°C for 1.0 min; annealing temperature as per Supplementary Table 1 for 1.0 min; extension at 72°C for 1.0 min, followed by one-cycle of 72°C for 5.0 min; and final cooling at 4.0°C.

Then, the respective PCR products were resolved in 1.0% agarose gel followed by excision and elution using a gel elution kit according to the manufacturer’s instructions (Promega Corporation, Madison, WI, USA). The PCR products were subjected to bidirectional sequencing using Sanger’s dideoxy chain termination technique to obtain the maximum gene sequence coverage. Contigs were assembled with DNA-Baser v5,^3^ followed by curation with Finch TV,^4^ and BLAST analysis was used to compare them to GenBank sequences.^5^ All the gene sequences were assigned accession numbers and deposited in the NCBI GenBank database.

##### Molecular phylogenetic analysis

Using Mega X with 1000 bootstrap replications the Maximum Likelihood algorithm and Hasegawa-Kishino-Yano model were used to perform multi-gene sequence-based phylogenetic analysis (Felsenstein, 1985; Hasegawa et al., 1985; Kumar et al., 2018). The gene sequences of *Microbacterium* sp. isolated from the diverse natural environments were retrieved from NCBI databases (see text footnote 1). CLC Sequence Viewer was used to normalize the nucleotide sequences to a constant length of 767 to 959 base positions (CLC Sequence Viewer 8).

### Studies on endophytic colonization of *Microbacterium testaceum* D18 in rice plant

#### Genetic transformation of *Microbacterium testaceum* D18 with a reporter gene (green fluorescent protein)

A stable green fluorescent protein (GFP) gene was inserted into the genome of the *M. testaceum* D18 strain by the tri-parental mating method. Before the transformation, spontaneous rifamycin-resistant *M. testaceum* D18 was selected by plating on to nutrient agar medium amended with rifamycin (50 μg mL^–1^) and incubated for 48–72 h at 28°C. The rifamycin-resistant colony was subsequently used in the transformation experiment essentially to counter-select against *Escherichia coli* XL1 Blue in the conjugation experiment. We used a Tn7-based GFP construct “pBKminiTn7gfp2Gm10” maintained in *E. coli* XL1 Blue (Gentamycin 20 g mL^–1^) and a helper plasmid “pUXBF13Amp100” maintained in *E. coli* XL1 Blue (Ampicillin 100 g mL^–1^) to tag the bacteria. The tri-parental-mating was conducted to insert the GFP gene into the bacterial genome (Sheoran et al., 2015). The transformed colonies were selected on NA plates amended with Gentamycin (20 μg mL^–1^) and Rifamycin (50 μg mL^–1^) incubated at 28°C for 48–72 h.

Putatively transformed colonies that appeared on the antibiotic-amended selection plates were sub-cultured on fresh NA plates amended with the same antibiotics. The colonies that appeared were then subjected to PCR confirmation for the insertion using GFP-specific forward and reverse primers (Ashajyothi et al., 2020). Briefly, primer pairs gfp-Rt-F-5′GGCCGATGCAAAGTGCCGATAAA3′ and gfp-Rt-R-5′AGGGCGAAGAATCTCGTGCTTTCA3′ were used in PCR reaction using GoTaq PCR kit of Promega (GoTaq Buffer–1X, DMSO–6%, dNTPs–200 mM, MgCl_2_–1.5 mM, forward/reverse primers–10 picomoles each, Taq polymerase–1 U) at an initial denaturation at 95°C for 5 min, 35 cycles of denaturation at 95°C for 1 min, annealing at 53°C for 30 s and extension at 72°C for 30 s followed by a final extension at 72°C for 5 min. In a 2% agarose gel, a 142-bp PCR amplicon was observed and documented (Quantity One Image Analysis system, Bio-Rad, CA, USA). The colonies that yielded expected amplicons in PCR-reaction were image analyzed under a confocal microscope [confocal laser scanning microscopy (CLSM) DM6000, Leica Microsystems, Wetzlar, Germany] for checking the stable expression of the green fluorescent protein. Further, the transformed bacterial strain with stable expression of gfp was preserved as glycerol stock for downstream activities and designated as *M. testaceum* D18::gfp.

#### Assay for endophytism

Endophytic colonization was studied using genetically tagged *M. testaceum* D18::gfp in the rice cv. Pusa Basmati-1. *M. testaceum* D18::gfp was cultured on double antibiotic-amended NA plates as described earlier. Surface-sterilized seeds of rice were soaked in a bacterial suspension of 10^8^ CFU mL^–1^ (1.0 OD at A_600_) for 24 h and allowed to germinate and grow in Petri dishes incubated under greenhouse conditions (RH > 90% and temperature 28–30°C) for 7-days. Seeds soaked in sterile double distilled water served as mock. The saplings were, then, transplanted into small pots with sterilized farm soil and grown under greenhouse conditions.

#### Confirmation of endophytism using confocal laser scanning microscopy

*Microbacterium testaceum* D18::gfp inoculated 1-month-old rice plantlets were taken, and thin slices of various plant parts were fixed in *p*-formaldehyde (4.0%) for 12 h at 4°C. Leaf, stem, and root cross-sections were scanned, and imaged at multiple sites using CLSM (DM6000, Leica Microsystems, Wetzlar, Germany). The images were processed and examined to determine bacterial localization inside the plant tissue.

#### Detection by PCR using green fluorescent protein-specific PCR markers

PCR-based test of the endophytic bacteria *M. testaceum* D18::gfp was performed using gfp-specific primers (Rt F-5′GGCCGATGCAAAGTGCCGATAAA3′ and Rt R-5′ AGGGCGAAGAATCTCGTGCTTTCA3′). For the tissue colonization study, the root, shoot, and leaves of the treated plants were crushed separately and 1 mL water extract of each tissue was centrifuged to get the pellet. The resulting pellet was used for the isolation of total genomic DNA using the protocol of bacterial DNA isolation as described earlier. The composition of the reaction mixture, as well as reaction conditions, was the same as that used for the selection of gfp transformants. The PCR amplicon was separated on agarose gel amended with ethidium bromide and visualized with a UV *trans*-illuminator (BioRad Laboratories, CA, USA).

### Characterization of plant probiotic features assay

#### Mineral solubilization assay

*Microbacterium testaceum* D18 was investigated for a range of plant probiotic characteristics. All experiments were done in triplicates with 10 μL bacterial suspension in sterile water as treatment and with appropriate control (10 μL sterile water without bacteria). Phosphate solubilization activity was accessed using an agar medium containing tricalcium phosphate (CaP; 3 gL^–1^), as the method described by Chen et al. (2006). The method described by Aleksandrov et al. (1967) was used to determine the potassium (K) solubilizing activity by using an agar medium containing inorganic potassium source Potassium Aluminosilicate (AlKO_6_Si_2_). Similarly, the solubilization of mineral zinc was detected in a modified Pikovskaya agar medium according to Pikovskaya (1948) containing insoluble zinc sourced from ZnO. The ability to produce siderophore was assayed by adopting the method described by Louden et al. (2011) using CAS agar medium as substrate.

#### Indole-3-acetic acid and ammonia production

The technique described by Patten and Glick (1996) was used to test indole-3-acetic acid (IAA) production using L-tryptophan in DF salt minimal medium and Salkowski et al.’s (1996) reagent. The ability to produce ammonia was assayed in Nutrient Broth after adding Nessler’s reagent as described by Zhou and Boyd (2016).

#### Enzymes assays

*Microbacterium testaceum* D18 was also analyzed for its ability to produce a range of defense-related enzymes under *in vitro* conditions. By using the agar well diffusion method, the bacterium *M. testaceum* D18 was inoculated on casein agar, carboxymethyl cellulose (CMC) agar, starch agar, pectin agar, and xylan agar medium to evaluate the production of protease, cellulase, amylase, pectinase, and xylanase, respectively (Ruiz et al., 2009).

Briefly, in the nutrient agar medium, 1.0% of each substrate was used to make substrate media plates. On the solidified media, a well with a diameter of 5.0-mm was made with a cork borer, and the bacterial cell suspension (10 μl of 0.5 OD_*A600*_) was added. The plates were then incubated for 48 h at 28°C. A zone of clearing around the wells was measured, and a minimum diameter of 15 mm was considered a positive result. For analysis of enzyme activity such as pectinase and amylase, plates were flooded with iodine–potassium iodide solution for 15 min and then treated for 10 min with sodium chloride (1.0 mol L^–1^) for de-staining.

By flooding the plates with Congo Red solution (1.0%) for 15 min and then de-staining with sodium chloride (1 mol L^–1^) for 10 min, cellulase and xylanase enzyme activity were identified. In casein agar, the formation of clear zones around the wells was utilized to measure the protease production. To assess the production of chitinase enzyme nutrient agar media amended with 1% (v/v) colloidal chitin was used and inoculated with bacterial suspension (5 μL) and incubated for 1 week. Later, the plates were flooded with Congo red (1%). The formation of an orange zone surrounding the colony was marked as positive.

### Antagonistic activity of *Microbacterium testaceum* D18 on *Magnaporthe oryzae in vitro*

#### Secreted metabolite-mediated antagonism

To assess the antagonistic activity of secretory metabolites released by *M. testaceum* D18 against the mycelial growth of *Magnaporthe oryzae* (hereafter *M. oryzae*), a dual culture confrontation assay was conducted in small Petri plates (40.0 mm). A modified TSA-PDA medium [Tryptic soy agar (SRL, Mumbai, India) Potato dextrose agar (HiMedia Laboratories, Mumbai, India)] was poured onto Petri plates and freshly grown mycelial disk (0.5 cm diameter) of the fungus was inoculated at one corner of the plate. The bacterial inoculum on the agar surface was streaked parallel to the mycelial disk and incubated at 28°C for 5–7 days. The mycelial growth was measured and compared against the mock plate to calculate the % inhibition zone. The experiment was performed twice with three replications each. For calculating the mycelial inhibition, the following formula was used.

Inhibition(%)=C-T/C× 100

where, C = Colony diameter in control, and T = Colony diameter in treatment.

#### Volatile compound mediated antagonism

*In vitro* antagonistic activity of bacterial volatile organic compounds released by *M. testaceum* D18 isolate was investigated on *M. oryzae*-1637 using the Sheoran et al. (2015) methodology. In the center of a PDA plate, a mycelial disk from a freshly grown fungal culture was placed. Similarly, another Petri plate poured with a Tryptic soy agar plate (HiMedia Laoratories, Mumbai, India) was inoculated with 20 μL of 10^8^ CFU mL^–1^ mid-log phase bacterial culture. To allow the fungus to be directly exposed to bacterial volatile, the lids of both plates were separated and the inoculated plates were placed face-to-face and fastened with parafilm, Petri seal tape, and klin film so that no volatile should escape from inside during the experiment. Three replications per treatment with proper control were incubated at 28°C for 5–7 days. Further, colony diameter was measured and % mycelial inhibition was calculated using the same formula.

### Chemo-profiling of bacterial volatile organic compounds

#### Analysis of bacterial volatile compounds by solvent extraction method

Under *in vitro* conditions, the volatiles generated by *M. testaceum* D18 were able to block fungal growth. This observation led to the investigation of the bacterial isolate’s volatile profile. Bacterial volatile organic compounds were extracted in HPLC grade hexane using the solvent extraction method (Joana Gil-Chávez et al., 2013) from a 72-h-old bacterial culture for this study. Hexane, which was used as a solvent and dissolved with bacterial organic compounds, was also analyzed using gas chromatography-mass spectroscopic (GC-MS).

#### Gas chromatography and mass spectroscopic conditions

An HP-5MS column (60 m 0.25 mm; /0.25 m, Thermo Co., USA) coupled to a triple-axis mass spectrometer (ThermoFisher Scientific, MA, USA) was used for GC-MS analysis. The injection volume was kept at 1.0 L with flow mode in split control. The flow rate of helium carrier gas was set at 1.0 mL min^–1^, with a head pressure of 10 psi. GC-MS was performed with the following condition; the oven temperature was maintained at 40°C for the first minute. The temperature was then raised to 120°C at an increasing rate of 3°C min^–1^ and then maintained for 2 min.

Finally, the temperature rose by 4°C min^–1^, reaching its maximum at 280°C. The movie lasted a total of 65 min. Electron ionization 70 eV, Ion source 180°C, full scan mode (50–550 mass units), E.M voltage 1376, transfer line temperature 280°C, and solvent delay 3.0 min, were the MS acquisition parameters. At a scanning time of 1.0 s and in the mass range of 50–550 AMU, the ionization energy was 70 eV. By comparing mass spectra, compounds were identified. The Mass Spectra Library of the National Institute of Standards and Technologies (NIST) was utilized as a reference for determining the fundamental components.

### Assessment of plant pathogenic nature of *Microbacterium testaceum* D18

#### Hypersensitive reaction on *Nicotiana tabacum*

Tobacco (*Nicotiana tabacum*) plants were grown in aclimate-controlled greenhouse. Two to 3-month-old plants with fully expanded 7–8 leaves were used in the experiment. Pre-inoculation conditioning was given to the plants under greenhouse conditions at a temperature of 20°C and 50–60% relative humidity and a 12 h light/dark period for 2.0-days. Fully expanded leaves that were attached to the plants were used in all experiments.

Most of the phytopathogenic bacteria are known to cause hypersensitive reactions (HR) on tobacco leaves which are visible as water-soaked necrotic lesions upon leaf infiltration (Klement, 1963). Similarly, most non-pathogenic bacteria will not cause any hypersensitive reaction. *M. testaceum* D18 on Nutrient agar and suspension of 1 × 10^8^ CFU/mL (1.0 OD at A_600 *nm*_) were prepared in sterile distilled water. It was infiltrated on potted *N. tabacum* leaves using a sterile hypodermal syringe. The plants were kept for incubation at 25–30°C under glasshouse conditions (12 h of dark/light photoperiods). Likewise, for negative control sterile double distilled water was infiltrated on leaves. Leaf infiltrated with plant pathogenic bacterium, *Ralstonia solanacearum*, served as a positive control. Induced responses of tobacco leaves were recorded after 24 h post-inoculation (hpi).

### Effect of *Microbacterium testaceum* D18 colonization on the growth of rice seedlings

Firstly, the seed priming assay was conducted using *M. testaceum* D18 to test the effect on germination %, root/shoot length, fresh weight, and dry weight in the rice, cv. Pusa Basmati-1 and BPT5404. The bacterium *M. testaceum* D18 was cultured on NA mixed with the two antibiotics described earlier. Rice seeds were surface sterilized and soaked in sterile distilled water for 24 h. Bacterial suspension of cell concentrations at 10^8^ and 10^7^ cells mL^–1^ were prepared using mid-log phase culture and seeds were allowed to grow on it for 7 days under green-house conditions (RH > 90% and temperature 28–30°C). The observations were taken for germination %, shoot length, root length, number of roots, fresh weight, and dry weight at 7 days post-inoculation. For mock, seeds were incubated only on sterile deionized water. The experiment was performed in three replications with 20 seeds each and repeated twice.

### Evaluation of *Microbacterium testaceum* D18 for blast disease suppression

Blast susceptible varieties, Pusa Basmati-1 and BPT5204 were seed bacterized with two cell densities 10^7^ and 10^8^ CFU mL^–1^ (0.1 OD and 1.0 OD at A_600_, respectively) of bacterial suspension and allowed to germinate for 5 days. After germination, the seedlings were transplanted into small two-inch pots with sterile farm soil and grown for 18–21 days till they attained 3-leaf stage under greenhouse conditions (28 ± 2°C with 85 ± 10% relative humidity and 14/12 h of light/dark cycle). *M. oryzae* was cultured on rice straw extract sucrose agar medium at 25°C for 7–8 days and was used for foliar inoculation in the experiment. 48 h before pathogen inoculation, a prophylactic spray of bacterial cell suspension was given above the foliage using a glass atomizer.

The conidial suspension (∼2 × 10^5^ conidia mL^–1^) amended with Tween 20 (0.05%) was prepared and sprayed over the foliage of 3-week-old seedlings (Rajashekara et al., 2017). Seedlings kept for positive control were not sprayed with bacteria while plants sprayed with tricyclazole (0.1%) served as a positive control. Further, plants were incubated for conditioning in the dark for 24 h at 22°C ± 2°C with 90% RH in a climate-controlled greenhouse. After that plants were kept for 7–10 days under greenhouse conditions with a 12 h dark/light cycle, temperature 22°C ± 2°C, 90% RH with leaf wetness maintained by spraying water three times a day.

The severity of the blast was measured 7–10 days post-inoculation using a 0–5 disease rating scale as described by Mackill and Bonman (1992). The disease scores were used for the calculation of disease severity using the following formula.

Disease ⁢severity=∑(Scale×Number⁢of⁢plants⁢infected)⁢×⁢100Total⁢number⁢of⁢plants×Maximum⁢disease⁢scale

Likewise, the % reduction in disease severity against control was calculated using the following formula.

$$\mathrm{Reduction}\,\mathrm{in}\,\mathrm{Blast}\,\mathrm{Severity}=\frac{\text{C}-T}{\text{C}}\times 100$$

where C = Disease Severity in control, and T = Disease Severity in treatment.

### Transcriptional analysis of immunocompetence induced by bacteria

Following confirmation of the blast suppression capacity, qPCR tests were carried out to determine the influence of bacterial supplementation on the expression of genes implicated in rice defense pathways.

Briefly, germinated seeds of Pusa Basmati-1 as well as BPT5204, each bacterized with 10^7^ and 10^8^ CFU mL^–1^ bacterial suspension separately were sampled after 7 days post bacterization at the seedling stage. Further, the seedlings were planted in small pots with sterile soil. At the three-leaf stage, a booster dose of respective bacterial suspension (same concentration as seed treatment) was given and sampling was done after 24 h (before challenge inoculation by a pathogen). Samples were quickly snap-frozen in liquid nitrogen to cease all cellular metabolic activity and then kept at −80°C until needed. The SV Tool RNA isolation system was used to isolate total RNA according to the manufacturer’s instructions (Promega Corporation, Madison, WI, USA). NanoDrop 2000 (Thermo Scientific, MA, USA) and agarose gel electrophoresis were used to determine the quantity and quality of RNA.

Rice genes associated with defense-related pathways such as *OsCERK1* (Kouzai et al., 2014), *OsFMO1* (Koch et al., 2006; Mishina and Zeier, 2006), *OsCEBiP* (Akamatsu et al., 2013), *OsNPR1* (Sugano et al., 2010), *OsPAD4* (Ke et al., 2014) and *OsEDS1* (Ke et al., 2019), playing a key role in rice innate immunity were selected (Supplementary Table 2). qPCR primers for the above defense genes are given in Supplementary Table 3. qPCR was performed using GoTaq^®^ 1-Step RT-qPCR System (Promega Corporation, Madison, WI, USA) in Real-Time Thermal Cycler (Light Cycler 96, Roche Life Science, Penzberg, Germany).

The following were qPCR reaction conditions; one step of reverse transcription at 37°C for 15 min followed by reverse transcriptase inactivation step of 95°C for 10 min followed by 30 cycles of 95°C for 10 s, annealing at 58°C for 30 s, and extension at 72°C for 30 s followed by three-step melting of 95°C for 10 s, 63°C for 60 s, and 97°C for 1.0 s and then final cooling at 37°C for 30 s. Later, cyclic threshold data points were analyzed for the determination of gene expression relative to reference housekeeping *OsActin* gene using the software LightCycler^®^96 Roche. The mean Ct values were considered for the calculation of 2^–ΔΔCT^ to estimate the fold changes in gene expression.

### Statistical analysis

Using the statistical tool GraphPad Prism 9,^6^ all of the experimental data with good biological and technical replications were pooled and analyzed for the one-way analysis of variance (ANOVA) and two-way ANOVA at *p* ≤ 0.05 (*), *p* ≤ 0.01 (**), and *p* ≤ 0.001 (***) levels of significance with *Post-hoc* analysis. Furthermore, the statistical analyses for qRT-PCR data were performed using WASP-Web Agri Stat Package 2.0 to group the significant results into different clusters. The data sets with the same alphabet were significantly similar to each other while significantly non-similar data sets were designated with different alphabets. The ns denotes non-significant.

## Results

### Characterization of bacterial isolate OsEnb-ALM-D18

Rice endophytic bacterial isolate OsEnb-ALM-D18 which was identified as *M. testaceum* D18 by 16S rRNA gene sequencing was used in the present study. Firstly, the identity of the isolate was reconfirmed by using multiple approaches to support the 16S rRNA gene sequencing data. On nutrient agar media *M. testaceum* D18 appeared as typical small round, convex, on-fluid yellow colonies while in NA added with 2,3,5-triphenyl tetrazolium chloride (TTC) it appeared with the same shape and size of colonies but are bright red (Supplementary Figure 1A). The identification of the endophytic bacterium was again confirmed through TEM imaging (Supplementary Figure 1B) which indicated the different structures like oval, rod, and V shape structures typical of *Microbacterium*.

### Multigene sequencing, identification, and phylogenetic analysis

**FIGURE 1:**
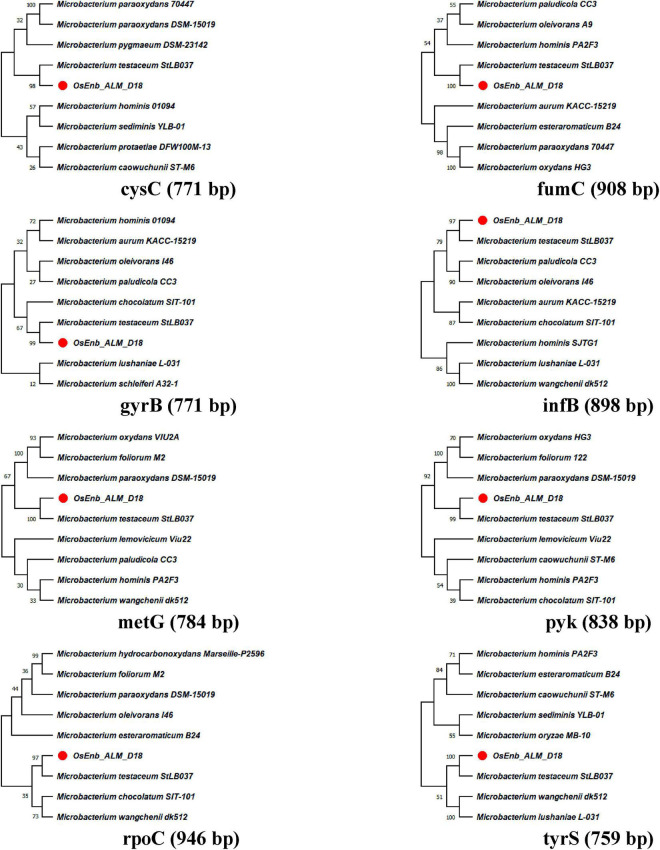
Molecular phylogenetic analysis of *Microbacterium* species by maximum likelihood method. The evolutionary history was inferred by using the Maximum Likelihood method and the Hasegawa-Kishino-Yano model (Hasegawa et al., 1985). The % of trees in which the associated taxa clustered together is shown above the branches. This analysis involved the nucleotide sequence of nine isolates. Evolutionary analyses were conducted in MEGA11 (Kumar et al., 2018).

A total of eight genes of *Microbacterium* were used for phylogenetic analysis as well as identity confirmation of the rice endophyte OsEnb-ALM-D18. nBLAST results of the different genes are presented in Supplementary Figure 1C; Table 1 where all of the gene sequences identify the isolate as *M. testaceum*. Further, all the gene sequences were submitted to NCBI GenBank. Further, the accession numbers were assigned (Accession numbers ON157417-ON157424). However, phylogenetic analysis was performed for eight gene sequences with the sequences of closely related bacterial species obtained from NCBI GenBank databases further confirming the identity of rice endophyte OsEnb-ALM-D18 as *M. testaceum* with more than 97% bootstrap value (Figure 1; Table 1; Supplementary Table 4).

**TABLE 1:** Multigene sequences based identity of endophytic *Microbacterium* by closest match.

Gene name	Sequence length (bp)	Max score	Total score	Query cover (%)	*E*-value	Identity (%)	*Closest match	Species identity	Accession number assigned
D18_cycS	808	953	953	100	0.0	88.00	*M. testaceum* StLB037	*M. testaceum*	ON157417
D18_fumC	910	1,299	1,299	100	0.0	92.42	*M. testaceum* StLB037	*M. testaceum*	ON157418
D18_gyrB	788	1,112	1,272	100	0.0	92.13	*M. testaceum* StLB037	*M. testaceum*	ON157419
D18_infB	898	1,448	1,448	100	0.0	95.77	*M. testaceum* StLB037	*M. testaceum*	ON157420
D18_metG	785	1,212	1,212	100	0.0	94.52	*M. testaceum* StLB037	*M. testaceum*	ON157421
D18_pyk	846	1,303	1,303	100	0.0	94.44	*M. testaceum* StLB037	*M. testaceum*	ON157422
D18_rpoC	946	1,637	1,637	100	0.0	97.89	*M. testaceum* StLB037	*M. testaceum*	ON157423
D18_tyrS	767	1,157	1,157	100	0.0	93.87	*M. testaceum* StLB037	*M. testaceum*	ON157424

*Gene sequences were BLAST analyzed in GenBank database https://blast.ncbi.nlm.nih.gov/Blast.cgi?PROGRAM=blastn&PAGE_TYPE=BlastSearch&LINK_LOC=blasthome. Closest match as of October 2022.

### Transformation of *Microbacterium testaceum* D18 expressing the reporter gene, green fluorescent protein

To assay the endophytism of *M. testaceum* D18, firstly, intrinsic rifamycin antibiotic resistance of the bacterium was exploited to select the colonies resistant to rifamycin. These resistant colonies were then used for gfp transformation using the triparental mating method and final transformed cells were selected on a double antibiotic (gentamycin + rifamycin) amended plates (Supplementary Figures 2A,B). The clonal nature of resistant colonies was further confirmed with the help of BOX-PCR fingerprinting and an identical amplicon profile was obtained (Supplementary Figure 2C).

Over 50 putative transformed colonies have found on the double antibiotic selection plates which were again confirmed with PCR as well as CLSM (Supplementary Figure 3). The colonies which showed a high level of gfp expression and were positive for gfp amplification were preserved as glycerol stock and used for downstream experiments. Transformed *M. testaceum* D18::gfp expressed gfp and showed resistance against gentamycin as well as rifamycin. Antibiotic resistance of the *M. testaceum* D18::gfp was used in the estimation of the endophytic population in the rice endogenous tissue.

### Endophytism of *Microbacterium testaceum* D18::green fluorescent protein in rice

#### Fluorescence microscopy

Plantlets of rice cultivar Pusa Basmati-1 emerged from seeds inoculated with *M. testaceum* D18::gfp (1.0 OD at A_600_) and were washed thoroughly in sterile distilled water and thin sections were fixed in 4% paraformaldehyde at 4°C for 12 h. Finally, the tissue scanning and imaging were performed in the confocal laser scanning microscope (CLSM; DM6000, Leica Microsystems) at different sites on the sections. The endogenous colonization of *M. testaceum* D18::gfp was confirmed in rice roots, stems, and the leaf using CLSM imaging of rice seedlings.

#### PCR-based confirmation of green fluorescent protein gene

**FIGURE 2:**
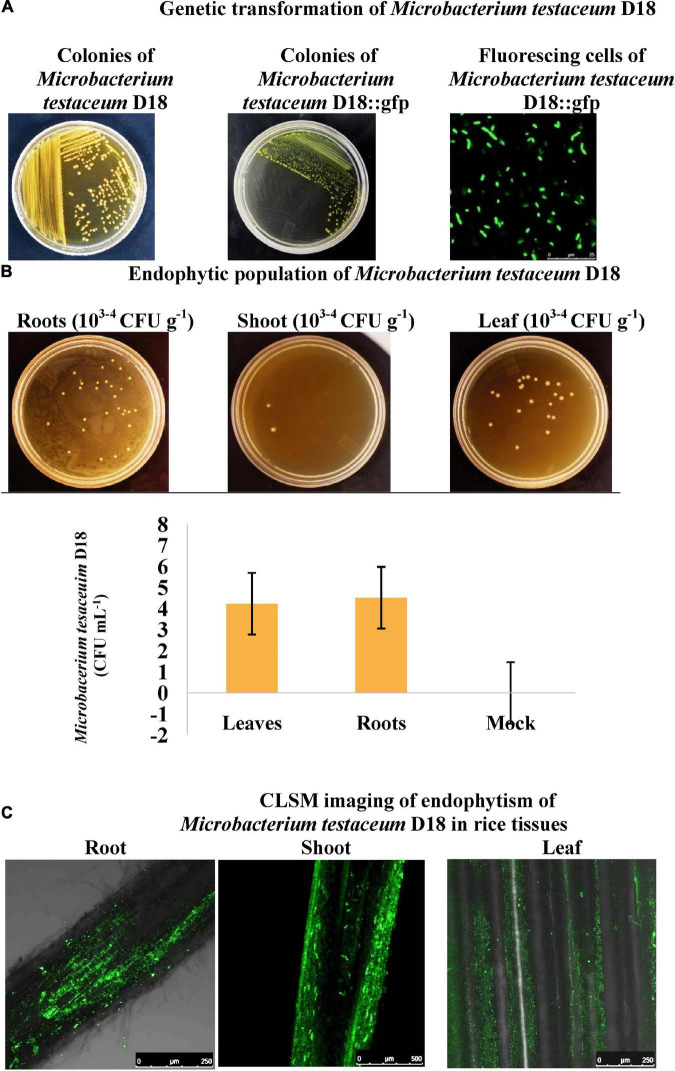
Endophytism of *Microbacterium testaceum* D18::gfp in rice plants as revealed by colony count method and confocal laser scanning microscopy. **(A)** Genetic transformation of *M. testaceum* D18 using Tn7-based neutral integration of gfp gene. **(B)** Plant count method exploiting gentamycin and rifamycin resistance conferred by Tn7-based vector (pBKminiTn7gfp2Gm10). **(C)** CLSM imaging of endophytically colonized *M. testaceum* D18::gfp.

Further, PCR amplification using gfp gene-specific primers resulted in 142 bp amplicon in 30 days old rice seedlings that emerged from bacterized seed that confirmed the endophytic nature of *M. testaceum* D18::gfp (Figure 2). Amplification was not observed in untreated rice seedlings.

### Characterization of plant probiotic features

**FIGURE 3:**
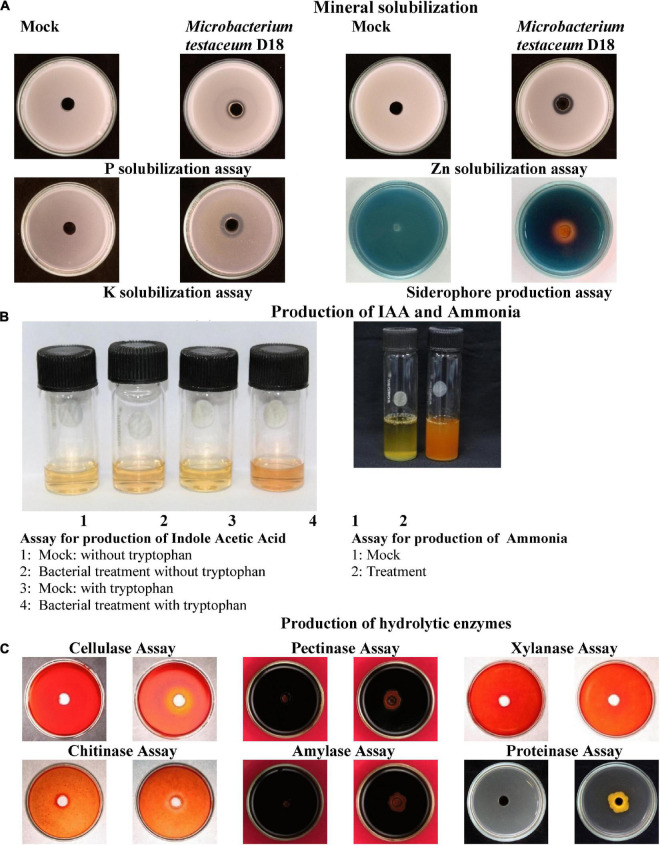
Plant probiotic features of *M. testaceum* D18. **(A)** Mineral solubilization assays. **(B)** Production of IAA and Ammonia. **(C)** Production of hydrolytic enzymes.

Plant probiotic nutrient solubilization and production of hydrolytic enzymes, siderophore, and phytohormone were tested. *M. testaceum* D18 tested positive for solubilization of phosphorus, potassium, and zinc, and production of siderophore, ammonia, and IAA. In the case of enzymatic activity, the bacterial isolate tested prolific for cellulase and chitinase production and negative for pectinase, xylanase, amylase, and protease (Figure 3; Supplementary Table 5).

### Antagonistic activity of *Microbacterium testaceum* D18 on *Magnaporthe oryzae*

**FIGURE 4:**
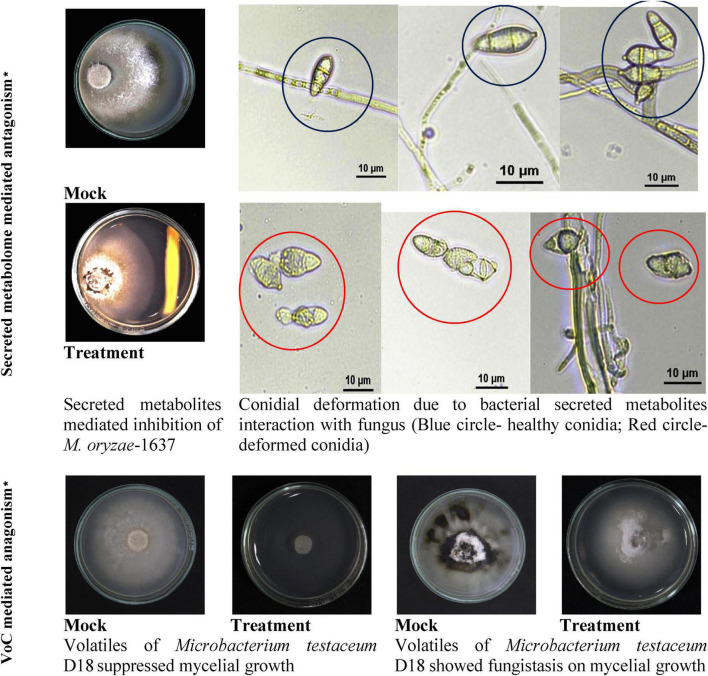
Secreted and volatile metabolite mediated antagonism of *M. testaceum* D18 on *M. oryzae*-1637. *% reduction in mycelial growth of *Magnaporthe oryzae* was observed over mock in both secreted metabolite as well as volatile mediated dual culture assays. In addition to this deformation in conidial morphology was observed in the secreted metabolite-mediated assay.

Endophytic bacterium *M. testaceum* D18 was found to inhibit the mycelial growth of *M. oryzae* primarily by its volatilome. Here, the volatile compounds showed 95.5% mycelial inhibition as against 29.9% inhibition by secreted metabolome. Further, the microscopic examination of *M. oryzae* conidia after bacterial exposure revealed abnormal structure compared to the unexposed conidia in mock suggestive of antibiotic effect on conidial development. However, the volatilome was found to be fungistatic rather than fungicidal as the mycelial growth re-emerged after the removal of volatile stress (Figure 4).

### Gas chromatography-mass spectroscopic profiling of bacterial volatile organic compounds produced by *Microbacterium testaceum* D18

After observing the inhibitory activity of the volatilome of *M. testaceum* D18 on rice blast fungus, the volatiles was extracted and analyzed using the solvent extraction method followed by fractionation in GC-MS. The gas chromatography showed the abundance of a wide variety of compounds in the volatilome of *M. testaceum* D18. A total of 11 chemical compounds were detected in the hexane extract of volatilome. Two compounds namely, 9-octadecenoic acid (32.53%) and hexadecanoic acid (30.06%) contributed over 60% of the chemicals of the volatilome. Other compounds were in the range of 0.26–5.7% in the volatile (Supplementary Figure 4; Supplementary Table 6).

### Assessment of plant pathogenic nature of *Microbacterium testaceum* D18

To test this tobacco hypersensitivity assay was conducted on intact tobacco leaves. *M. testaceum* D18 did not induce necrotic responses in tobacco leaves even after 48 h of leaf infiltration. However, the leaf infiltrated by the pathogen *R. solanacearum* showed prominent signs of rapid necrosis owing to the hypersensitive reaction, suggesting that *M. testaceum* D18 is potentially non-pathogenic in plants (Supplementary Figure 5).

### Effect of *Microbacterium testaceum* D18 colonization on seedling’s growth

**FIGURE 5:**
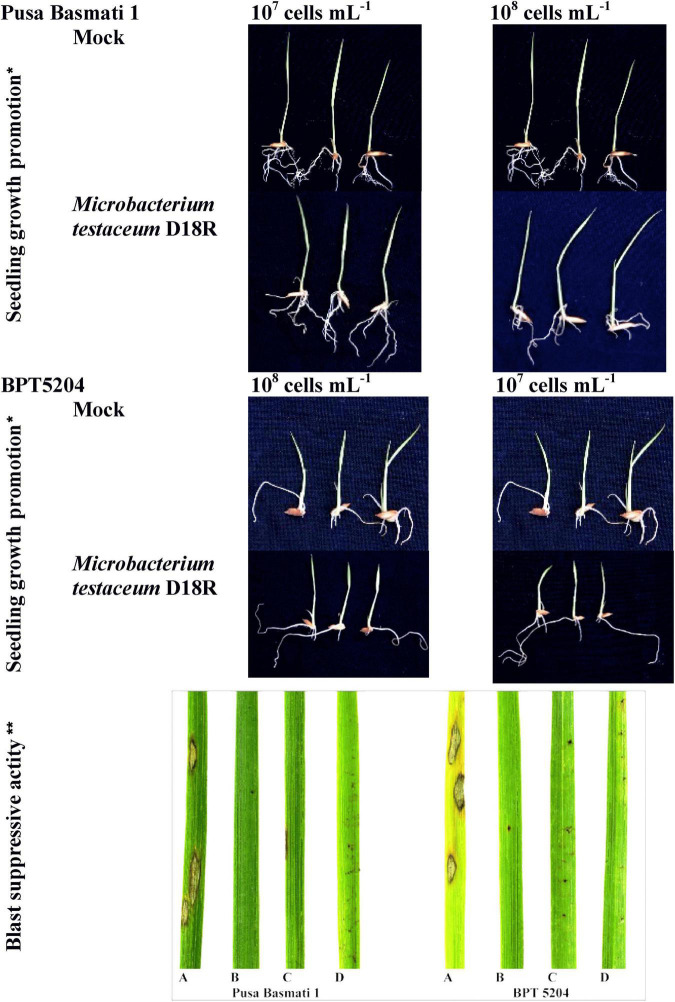
*Microbacterium testaceum* D18 induced growth promotion in rice seedlings, and blast disease suppressive activity. *M. testaceum stimulated the growth of rice seedlings upon bacterization; **M. testaceum D18 triggered a hypersensitivity type of reaction instead of large lesions observed in mock; the hypersensitive reaction indicates over-expression of defense genes. The number of lesions and size of lesions were found reduced in bacterized plantlets; plant responses are scored as per Mackill and Bonman. (A) Mock. (B) *M. testaceum* D18 (10^8^ CFU mL^–1^). (C) *M. testaceum* D18 (10^7^ CFU mL^–1^). (D) *D. Tricyclazole*.

The germinated seedlings on bacterial suspension were taken for observation. In the case of shoot length, 10^8^ CFU mL^–1^ on PB1 was found to increase significantly while in other treatments there was no difference observed. However, maximum root length was observed at 10^7^ CFU mL^–1^ on BPT5204 whereas the average number of roots was similar in all treatments. The fresh weight, dry weight as well as net weight in all treatments was found to increase slightly in all treatments (Figure 5; Table 2).

**TABLE 2:** Growth stimulant and blast suppressive activity of *Microbacterium testaceum* D18 on rice.

(A) Pusa Basmati-1									
Bacterial dose (CFU mL^–1^)	Germination (%)	*Shoot length	Root length	Number of roots	Fresh weight	Dry weight	Net weight	**Disease severity (%)	***Reduction in disease severity (%)
Mock	100	4.767^b^	4.233^b^	3.667	0.36^c^	0.145^b^	0.216^c^	**48.03**	–
10^8^	100	5.467^a^	3.467^c^	3.333	0.397^b^	0.153^a^	0.244^b^	**8.48**	**82.34**
10^7^	100	4.1^b^	4.467^a^	3.333	0.447^a^	0.152^a^	0.294^a^	**7.85**	**83.67**
**CD (0.05)**	**NS**	**0.149**	**0.115**	**NS**	**0.002**	**0.002**	**0.002**		
**CV**		**1.56**	**1.424**	**23.705**	**0.208**	**0.643**	**0.47**		

**(B) BPT5204**									

**Bacterial dose (CFU mL^–1^)**	**Germination (%)**	*Shoot length (cm)	**Root length (cm)**	**Number of roots**	**Fresh weight (g)**	**Dry weight (g)**	**Net weight (g)**	**Disease severity (%)	***Reduction in disease severity (%)

Mock	100	3.067	4.133^c^	2.667	0.281^b^	0.106^a^	0.175^b^	**45.71**	–
10^8^	100	3.033	4.4^b^	3.0	0.287^b^	0.099^b^	0.188^b^	**6.61**	**85.55**
10^7^	100	3.1	5.467^a^	3.667	0.32^a^	0.105^a^	0.214^a^	**7.58**	**83.41**
**CD (0.05)**	**NS**	**NS**	**0.149**	**NS**	**0.013**	**0.001**	**0.014**		
**CV**		**2.431**	**1.597**	**23.958**	**2.175**	**0.674**	**3.655**		

*Microbacterium testaceum D18 induced growth alteration in rice seedlings treated with 10^8^ and 10^7^ CFU mL^–1^, OD bacterial suspension after 7 days.

**Disease severity was calculated using the following formula: $\mathrm{Disease}\,\mathrm{severity}=\frac{\sum \left(\text{Scale}\times \mathrm{Number}\,\mathrm{of}\,\mathrm{plants}\,\mathrm{infected}\right)\times 100}{\mathrm{Total}\,\mathrm{number}\,\mathrm{of}\,\mathrm{plants}\times \mathrm{Maximum}\,\mathrm{disease}\,\mathrm{scale}}$.

***Blast severity reduction was estimated using the following formula: $\mathrm{Blast}\,\mathrm{severity}\,\mathrm{reduction}=\frac{\text{C}-\text{T}}{\text{T}}\times 100$ C = disease severity in control.

T = disease severity in treatment.

The different letters are statistically significant. Bold values represent the statistical parameters, critical difference, and coefficient of variance.

### Blast suppressive effect of *Microbacterium testaceum* D18

Rice cultivars Pusa Basmati-1, and BPT-5204 were used as susceptible checks for elucidating the blast disease suppressive effects of *M. testaceum* D18 at 10^7^ and 10^8^ CFU mL^–1^ applied as seedling treatment as well as a prophylactic foliar spray. The blast disease severity and incidence were scored according to the blast score chart recommended by Mackill and Bonman (1992) (Supplementary Figure 6). The bacterial isolate *M. testaceum* D18 suppressed blast disease in rice cultivars at tested doses. A significantly less blast severity score was observed at 10^8^ CFU mL^–1^ on both the varieties PB1 (8.48% PDI) and BPT5204 (6.61% PDI) as compared to untreated plants (45.71–48.03% PDI on PB1 and BPT5204). An over 82% reduction in blast severity over the mock was found in all the treatments (Figure 5; Table 2).

### qPCR assay on *Microbacterium testaceum* D18 conferred immunocompetence

**FIGURE 6:**
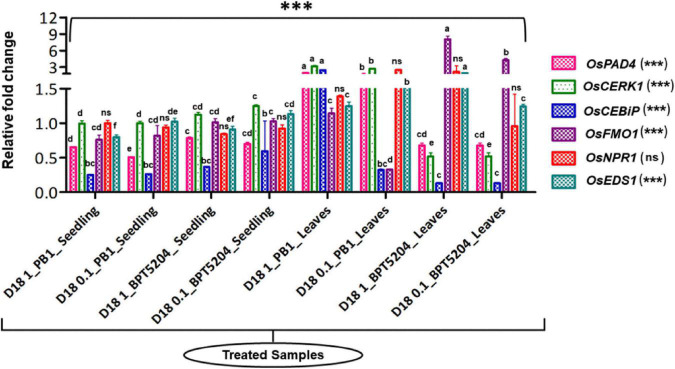
Transcriptional response of defense genes in rice upon bacterization by *M. testaceum*. qPCR-based transcriptional analysis of defense gene expression in rice seedlings upon endobacterization. The fold change values obtained for the defense genes were imported into the GraphPad Prism program (https://www.graphpad.com/scientific-software/prism), and two-way ANOVA was conducted using Bonferroni *post-hoc* test for determining the statistical significance at *p > 0.05, **p ≤ 0.001, and ***p ≤ 0.0001. Refer to Supplementary Table 7 for data pertaining to fold changes in gene expression. Different letters represent the different significant groups, based on completely randomized group test done using *p*-values.

Bacterized rice seedlings were subjected to a gene expression study by qPCR. Several defense-related genes such as *OsCERK1*, *OsCEBiP*, *OsNPR1*, *OsPAD4*, *OsEDS1*, and *OsFMO1* (Supplementary Table 2) showed a minimal to a high level of induction in bacterized rice seedlings compared with the mock where the seedlings were not treated with bacteria and *OsActin* was used as reference genes in this study. *Post-hoc* test confirmed that the bacterization led to a significant increase in the expression pattern of defense-related genes (at *P* ≤ 0.05 or *P* ≤ 0.01 or *P* ≤ 0.001) in both rice genotypes over mock plants. The expression of defense genes was even more pronounced after the booster dose application of bacteria on the phyllosphere. Maximum fold upregulation was observed for the *OsFMO1* gene after booster dose application at 10^8^ CFU mL^–1^ on BPT5204. Similarly, *OsCERK1* was maximally expressed in PB1 with 10^8^ CFU mL^–1^ foliar applications. Moreover, out of all assessed genes, *OsNPR1* was not significantly impacted by bacterial treatments, as analyzed by ANOVA. Besides, at the seedling stage, there was not much difference in the expression pattern of most of the genes while foliar application of 10^8^ CFU mL^–1^ was found effective in eliciting the expression of defense-related genes (Figure 6; Supplementary Table 7).

## Discussion

Fungal phytopathogen inciting crop diseases is a threat to agricultural production and food security in the world. Over the decades, two major strategies, i.e., resistance gene introgression and the use of fungicides, either alone or in combination have emerged as effective options for the management of fungal diseases. However, both strategies are inadequate for effective implementation owing to the emergence of new variants with renewed virulence (New Pathotypes) and resistance to fungicides [anti-microbial resistant (AMR)-Type]. The recent ban on fungicides for agricultural applications has created a void in commercial disease management in crop production systems. For instance, the ban on tricyclazole for rice blast management renewed the interest in other options for disease management. As a result, new strategies for combating fungal diseases in crop plants, particularly through the deployment of microbial biological control agents have gained acceptance (Sellem et al., 2017).

Here, we explored and evaluated endophytic bacteria isolated from rice leaves as antagonistic organisms of *M. oryzae* for biological control of blast disease. Phenotypically the *Microbacterium* colonies were tiny with pale yellow on nutrient agar medium. Microscopically the cells were small, short slender rods that occurred either in a single culture or in groups during the young stage. However, upon maturation, the cells become shorter and coccoid, and the ends of the cells are attached and produce a V-shaped appearance. The young cells are typically Gram-positive that upon maturation display variable Gram reactions. They are non-endospore producers, and often aflagellate or at times produce lateral flagella.

The species identity of the bacterium was confirmed as *M. testaceum* through16S rRNA gene sequence as well as multilocus sequence analysis (MLSA) (Fidalgo et al., 2016). For MLSA, we have used *Microbacterium*-specific eight genetic loci such as *rpoC*, *pyk*, *gyrB*, *tyrS*, *infB*, *cysS*, *metG*, and *fumC*. The endophytic *M. testaceum* isolated from rice leaf was found clustered with a potato leaf endophytic *M. testaceum* StLB037 (Morohoshi et al., 2009). Before the present study, *M. testaceum* was reported in diverse habitats like water (Epova et al., 2022), soil (Yang et al., 2014), tannery effluents (Elahi et al., 2019), phyllosphere (Sahu et al., 2021a,b), plant endosphere (Kumar et al., 2021b), and sometimes as an opportunistic pathogen in clinical samples (Leal et al., 2016). We further studied the endophytism of the bacterium in rice plants using gfp-tagged *M. testaceum* D18::gfp that could be visualized in the root, stem as well as leaf tissues.

Having established the species identity and confirmed the endophytism of *M. testaceum*, we have functionally characterized the bacterium for several traits such as plant probiosis, pathogen antibiosis; and blast suppressive activity *in planta*. The endophytic *M. testaceum* D18 promoted the growth of rice seedlings possibly through multipronged mechanisms such as phytohormone IAA production, solubilization of N, P, K, Fe, Zn, and secretion of hydrolytic enzymes as well as siderophores. Among them, IAA is an auxin that promotes cell proliferation, and elongation, and induces seedling growth (Verma J. P. et al., 2014). The data suggest that *M. testaceum* D18 promoted the availability of the major plant nutrients N, P, and K which contributed to growth promotion.

The production of ammonia is an important feature of biostimulants as ammonia production plays a key role in plant growth and development by contributing to N nutrition (Yadav et al., 2010; Mukherjee et al., 2020). P nutrition is needed for root development. In soil maximum proportion of phosphorus is unavailable owing to an insoluble state (Miller et al., 2010). Endophytic *M. testaceum* D18 showed P nutrition solubilization, -one of the key nutrients for plant growth. Previously, Misra et al. (2012) showed the ability of endophytes to solubilize the insoluble phosphate for enhancing leaf area and the consequently improved rate of photosynthesis (Reich et al., 2009). *M. testaceum* D18 was also found to solubilize K, the third most essential mineral nutrient for plant growth (Bahadur et al., 2016; Mukherjee et al., 2019).

In addition, *M. testaceum* D18 was also found to synthesize siderophores for iron-chelating Fe to make it unavailable to pathogenic organisms (Seong and Shin, 1996; Saha et al., 2016). Recent studies have suggested that siderophores can induce the defense mechanism of the plant via activating SA- and JA-signaling pathways (Aznar and Dellagi, 2015). In addition to siderophore, the Zn solubilization potential of the endophytic *Microbacterium* has been reported as a plant growth-promoting trait (Saravanan et al., 2004; Ali et al., 2018; Verma et al., 2022). Priming with *M. testaceum* D18 enhanced dry and fresh biomass, root and shoot length growth as well as their branching.

*Microbacterium testaceum* D18 secreted hydrolytic enzymes such as cellulase that are required during endophytic colonization and the endogenous spread (James et al., 2002; Lodewyckx et al., 2002; Walitang et al., 2017). Interestingly, *M. testaceum* D18 tested positive for other hydrolytic enzymes such as amylase, proteinase, and chitinase. Among them, the chitinolytic activity is particularly relevant as it potentially confers antifungal biocontrol traits against fungal pathogens like *M. oryzae* that contain chitin in the cell wall (Quecine et al., 2008). Our findings on probiotic features of *M. testaceum* D18 contribute toward increased shoot and root length, as well as enhanced biomass previously observed with other plant growth-promoting microorganisms (Verma P. et al., 2014; Mukherjee et al., 2020).

The rice endophytic *M. testaceum* D18 displayed excellent antifungal activity mediated majorly by volatile metabolome against rice blast fungus *M. oryzae* 1637. The effect of antifungal activities could be observed on mycelial growth and conidial structure. While the mycelia growth was inhibited due to the toxic effect of metabolites, the conidial shape appeared to be deformed. Having observed volatile mediated antifungal activity, we further characterized the volatilome of *M. testaceum* D18 by GC-MS revealing the high abundance of hexadecanoic acid and 9-Octadecenoic acid contributing to over 60% of the volatile chemicals. Among them, hexadecanoic acid emitted by *Trichoderma viride* has been shown to have antifungal activity against *Macrophomina phaseolina* (Khan et al., 2021). Apart from microbial origin, the hexadecanoic acid secreted by the plant *Sonchus oleracous* also showed antimicrobial properties (Banaras et al., 2020). 9-Octadecenoic acid (Oleic acid) has been the most abundant anti-bacterial unsaturated fatty acid naturally found in staphylococcal abscesses as well as on the skin surface (Speert et al., 1979; Dye and Kapral, 1981). The antimicrobial nature of other chemicals observed in *M. testaceum* D18 has been reported in the recent past; Tetradecanoic acid (Gajbhiye and Kapadnis, 2021; Nakkeeran et al., 2020), Propenoic acid butyl ester (Garnier et al., 2020), 2-Hexanone (Rocha et al., 2004), Trimethyl-heptadien-4-one (Kassam et al., 2021), 4-Dimethyl-1-heptene (Mannaa and Kim, 2018), 4-Methyl-2-pentanol (Petrović et al., 2013), and 3-Hexanone (Wang et al., 2020).

Blast suppressive activity of *M. testaceum* D18 was tested on seedlings that emerged from *Microbacterium* primed seeds of blast susceptible varieties PB1 and BPT5204 that showed significantly less disease severity as compared to untreated control plants. Previously, the biocontrol potential of endophytic *Microbacterium* StLB037 strains isolated from potato leaf against the soft rot pathogen *Pectobacterium carotovorum* subsp. *carotovorum* is reported (Wang et al., 2010). Recently Freitas et al. (2019) reported biological control of *Fusarium* root rot in cassava using *Microbacterium imperial* MAIIF2a. More recently Sahu et al. (2021a) showed a reduction in blast disease upon spraying of phyllosphere-adapted *Microbacterium oleivorans*. Other than the *Microbacterium* genus, antifungal bacterial genera such as *Achromobacter*, *Streptomyces*, and *Pseudomonas* have been reported against *M. oryzae* which reduced mycelial growth as well as disease development (Jha et al., 2011; Joe et al., 2012; Xu et al., 2017). Moreover, the perusal of records reveals that the present report on blast suppressiveness of endophytic *M. testaceum* appears to be novel.

The direct antagonism of the pathogen by endophytic *M. testaceum* D18, as well as the induction of defense-related pathways in the rice plant, could be responsible for disease suppression. Gene expression analysis using qRT-PCR revealed that *M. testaceum* D18 significantly upregulated the defense-related genes such as *OsFMO*, *OsCEBiP*, *OsNPR1*, *OsCERK1*, *OsEDS1*, and *OsPAD4*. Cultivar level difference was also observed between the two varieties. Notably, the highest level of induction was observed in the *OsFMO1* gene after bacterial suspension spray on BPT5204 plants. Similarly, PB1 sprayed with 10^8^ bacterial cells showed upregulation of all genes with the highest for *OsCERK1* (>3 fold). Among the genes, *OsCEBiP* and *OsCERK1* are receptor-like protein (RLP) and receptor-like kinase (RLK), respectively. Chitin and peptidoglycan perception has been reported for the activation of MAMP-triggered immune (MTI) responses in plants (Akamatsu et al., 2013; Kouzai et al., 2014). *OsPAD4* and *OsEDS1* genes are markers for jasmonic acid-mediated ISR in plants. *OsPAD4* is associated with the production of phytoalexin Momilactone-Ain rice plants (Hasegawa et al., 1985; Ke et al., 2014, 2019). Defense genes such as *OsNPR1* and *OsFMO* were marginally upregulated only after booster application, which is a key regulator of systemic acquired resistance mediated by salicylic acid (Sugano et al., 2010).

Having confirmed the plant probiotic, and pathogen antibiotic features as well as endophytism of *M. testaceum* D18, we performed a tobacco infiltration assay to determine the phytopathogenic nature, if any, and the consequent biological safety of the endophytic bacterium (Klement, 1963). *M. testaceum* D18 did not show any pathogenic reactions on tobacco suggestive of the non-pathogenic nature of the strain. Biosafety of plant-associated bacterium targeted for agricultural application as bioinoculants is of paramount importance (Kucheryava et al., 1999; Medina-Salazar et al., 2020; Sadeghi and Khodakaramian, 2020).

Taken together, it is concluded that rice endophytic *M. testaceum* D18 showed unique plant probiotic features as well as pathogen antibiotic activities. The endophytic strain suppressed rice blast disease similar to that of the fungicide, tricyclazole. Coupled with the ability to elicit host defense, the *Microbacterium* mediated biocontrol can be a supplementary strategy for blast disease management in rice farming. A perusal of published records revealed that this is the first report demonstrating the biocontrol and biostimulation activity of *M. testaceum* against blast disease.

## Data availability statement

The datasets presented in this study can be found in online repositories. The names of the repository/repositories and accession number(s) can be found in the article/Supplementary material.

## Author contributions

AP: conceptualization, methodology, data curation, formal analysis, investigation, writing—original draft, and fund acquisition. KPS: methodology, validation, formal analysis, and writing—original draft. SM: validation and writing—review and editing. AB, CK, TG, SK, and MA: validation. MK: validation and resources. NS and PN: methodology and supervision. AdK: methodology, resources, and supervision. AuK: conceptualization, methodology, formal analysis, resources, data curation, writing—review and editing, visualization, supervision, project administration, and funding acquisition. All authors listed have made a substantial and intellectual contribution to the manuscript writing, reviewing, and editing, have read and approved the publication of the manuscript in its current form.

## Footnotes

1
https://www.ncbi.nlm.nih.gov/search

2
https://www.bioinformatics.nl/cgi-bin/primer3plus/primer3plus.cgi

3
https://www.dnabaser.com/index.html

4
https://digitalworldbiology.com/FinchTV

5
https://blast.ncbi.nlm.nih.gov/

6
https://www.graphpad.com/
