# Supplementary material for: Rice leaf endophytic Microbacterium testaceum: Antifungal actinobacterium confers immunocompetence against rice blast disease
Source: Front Microbiol. 2022 Dec 21;13:1035602. doi: 10.3389/fmicb.2022.1035602 (PMC9810758; doi:10.3389/fmicb.2022.1035602)
Supplement: Supplementary file 1 [file Data_Sheet_1.docx]

| **Colonies of Rice Endophytes** | | **Close up view of colonies** | | | 1. Colony colour and pigmentation of *Microbacterium testaceum* D18 | |
| --- | --- | --- | --- | --- | --- | --- |
| Nutrient Agar | Nutrient agar +  2, 3, 5 triphenyl tetrazolium chloride | Nutrient Agar | | Nutrient agar +  2, 3, 5 triphenyl tetrazolium chloride |  |  |
| **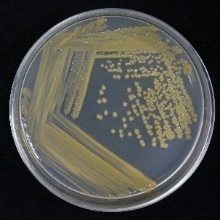** | **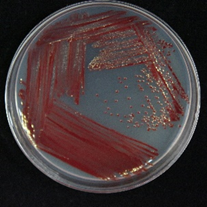** | **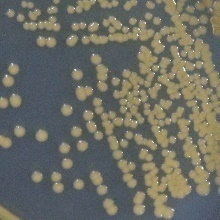** | | **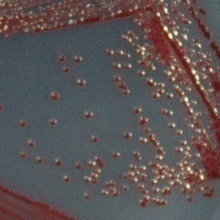** |  |  |
|  | | | | | | |
| 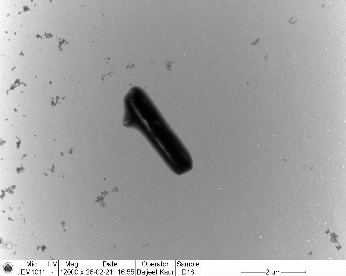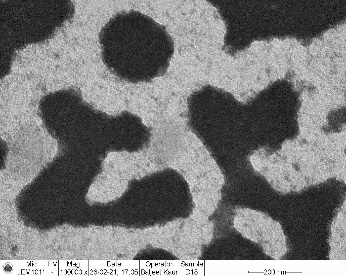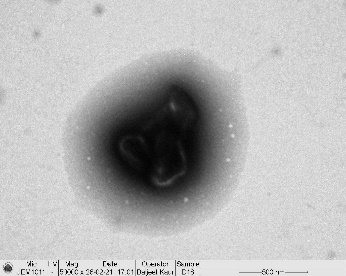   1. **TEM images of *Microbacterium testaceum* D18** | | | 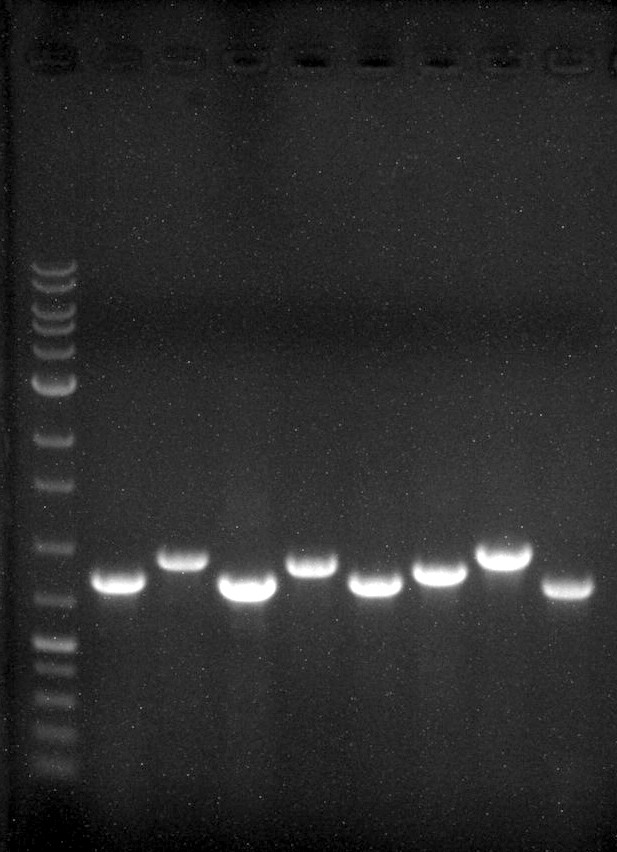 **M 1 2 3 4 5 6 7 8** | | | |
|  |  |  | 1. **Multigene amplicon profileof**   ***Microbacterium testaceum* D18** | | | |
|  |  |  | Lane M: 1 kb ladder  Lane 1: cycS (810 bp)  Lane 2: fumC (929 bp)  Lane 3: gyrB (795 bp)  Lane 4: infB (900 bp) | | | Lane 5: metG (793 bp)  Lane 6: pyk (858 bp)  Lane 7:rpoC (959 bp)  Lane 8:tyrS (767 bp)  Lane M: 1 kb ladder |

**Supplementary FIGURE 1|**Characterization and identification of endophytic *Microbacterium testaceum* D18.

| **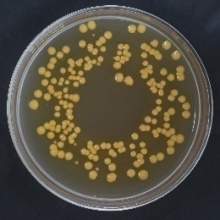** | **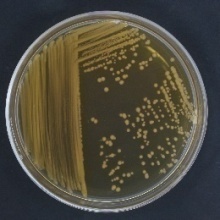** | 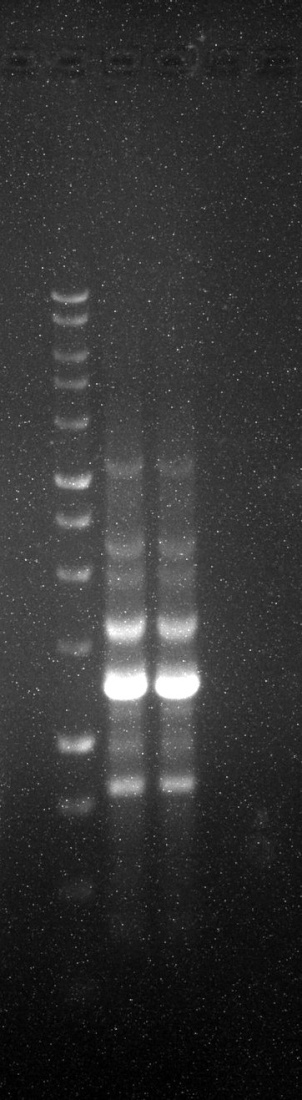 **M 1 2 3**M 1 2 | Lane M: 1 kb ladder; Lane 1: *Microbacterium testaceum* D18 (wild type); Lane 2: *Microbacterium testaceum* D18 |
| --- | --- | --- | --- |
| 1. **Selection of Rifamycin resistant type** | 1. **Confirmation of Rifamycin resistant type** | 1. **BOX-PCR-Based confirmation of genetic purity of rifamycin resistant types** | |

**Supplementary FIGURE 2|**Selection of rifamycin-resistant *Microbacterium testaceum* D18.

| **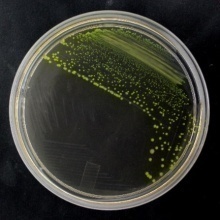** | **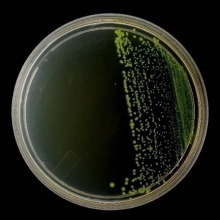** | 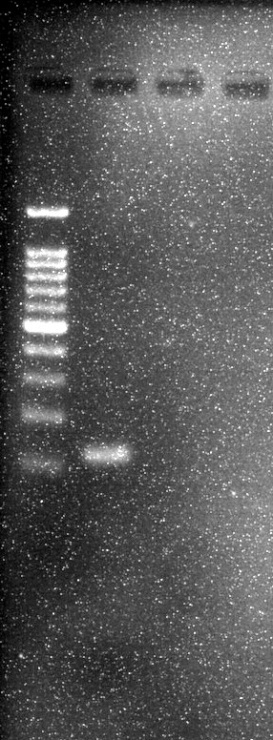 **M 1 2**  **PCR based detection using gfp- specific PCR markers**  Lane M: 1 kb ladder; Lane 1: *Microbacterium testaceum* D18::gfp; Lane 2: *Microbacterium testaceum* D18 |
| --- | --- | --- |
| ***Microbacterium testaceum* D18::gfp on NA (Rif50+Gm20)** | ***Microbacterium testaceum* D18::gfp on NA** |  |
| **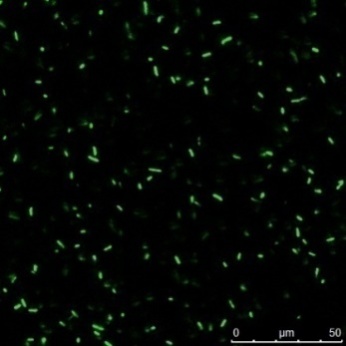** | **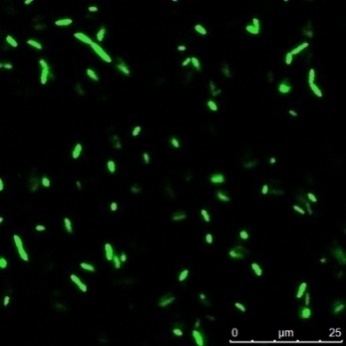** |  |
| ***Microbacterium testaceum* D18::gfp** | ***Microbacterium testaceum* D18::gfp** |  |

**Supplementary FIGURE 3|**Genetic transformation of *M. testaceum* D18 for gfp expression.

**Supplementary FIGURE4 |**Chemical profiling of *Microbacterium testaceum* D18 by GC-MS.

| **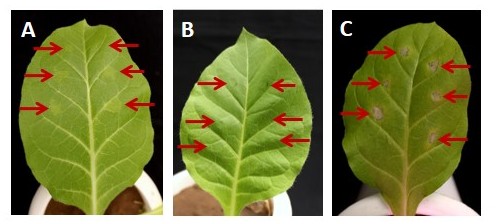** 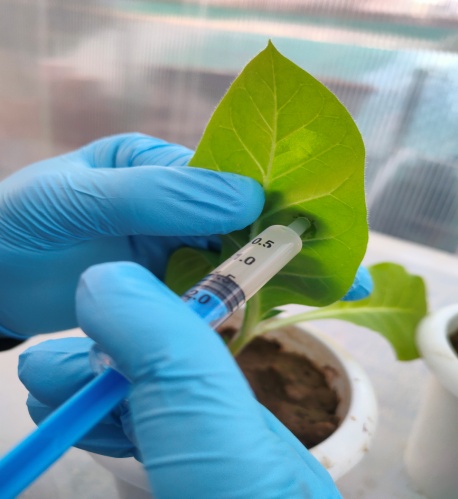 |
| --- |

**Supplementary FIGURE5 |**Assay for the pathogenic ability of *Microbacterium testaceum* D18by hypersensitivity assay on *N. tabacum****.*(A)**Leaf infiltrated with *M. testaceum* D18.**(B)** Leaf infiltrated with sterile distilled water (Negative control).**(C)** Leaf infiltrated with *Ralstonia solanacearum* (Positive control).

| 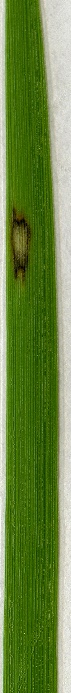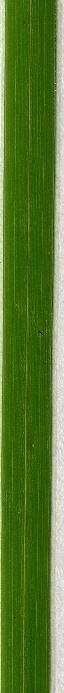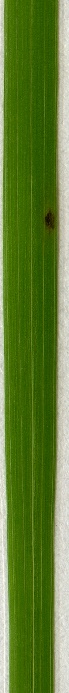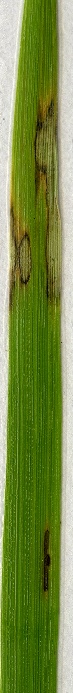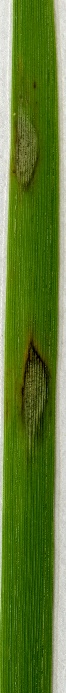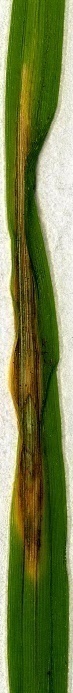 **0 1 2 3 4 5** |
| --- |

**Supplementary FIGURE6 |**Blast Disease scoring 0-5 scale given by Mackill and Bonman (1992). In here,0 = no evidence of infection; 1 = brown specks smaller than 0.5 mm in diameter; 2 = brown specks of 0.5–1.0 mm in diameter; 3 = roundish to elliptical lesions of about 1–3mmin diameter; 4 = typical spindle-shaped blast lesion, 3 mm or longer with little or no coalescence of the lesion; 5 = same as 4 but half or more leaves killed by coalescence of lesions.
