# Supplementary material for: Rice leaf endophytic Microbacterium testaceum: Antifungal actinobacterium confers immunocompetence against rice blast disease
Source: Front Microbiol. 2022 Dec 21;13:1035602. doi: 10.3389/fmicb.2022.1035602 (PMC9810758; doi:10.3389/fmicb.2022.1035602)
Supplement: Supplementary file 2 [file Data_Sheet_2.docx]

**Supplementary TABLE 1|**Rice defense genes used for the qPCR analysis and their function.

| **Gene** | **Characteristics and function** | **References** |
| --- | --- | --- |
| *OsCEBiP***  (Chitin Elicitor Binding Protein) | It is a Pattern Recognition Receptor (PRR) that detects the pathogen PAMP molecule chitin and activates the plant defense system. It is a plasma membrane protein that forms a receptor complex essential for fungal chitin-driven immune responses in rice. | Akamatsu et al. 2013 |
| *OsCERK1***  (Chitin Elicitor Receptor Kinase) | It is a rice receptor-like kinase (RLK) that mediates the signal of a fungal cell wall component chitin. It is indispensable for chitin perception and participates in innate immunity. It can mediate the signaling pathways of both fungal and bacterial PAMP molecules. | Kouzai et al. 2014 |
| *OsPAD4**  (Phytoalexin deficient 4) | Phytoalexin deficient 4 (PAD4) induces JA-dependent induced systemic resistance. It also plays an important role in the accumulation of JA and a terpenoid-type phytoalexin mamilactone A (MOA). | Ke et al. 2014 |
| *OsEDS1**  (Enhanced disease susceptibility 1) | Enhanced disease susceptibility 1 (EDS1) induces JA-dependent induced systemic resistance. | Ke et al. 2019 |
| *OsNPR1**  (Non-Repressor of Pathogenesis related Protein) | A central regulator of salicylic acid (SA)-mediated defense signaling  Reallocation of energy and resources during defense responses | Sugano et al. 2010 |
| *OsFMO1**  (Flavin-dependent Monooxygenase 1) | An essential component for induced systemic acquired resistance (SAR) | Mishina et al. 2006; Koch et al. 2006 |
| *Os*Actin | Housekeeping reference gene used in the analysis |  |

**Supplementary TABLE 2|**List of the PCR primers used in the gene expression studies.

| **Gene** |  | **Primer sequence (5’→3’)** | **Number of bases** | **Product size (bp)** |
| --- | --- | --- | --- | --- |
| ***OsCEBiP* | Forward | GTGCGGAGAAGTCTGGAAAG | 20 | 131 |
|  | Reverse | TCCTGATTTCGCTTGCTTTT | 20 |  |
| ***OsCERK1* | Forward | AAGAACTACCGGGCAAAGGT | 20 | 244 |
|  | Reverse | GCCCCTTTGAATCACTTGAA | 20 |  |
| **OsPAD4* | Forward | TGCCGACTACCACCGAAAC | 19 | 61 |
|  | Reverse | CCGGCCATGGGTGATGTA | 18 |  |
| **OsEDS1* | Forward | TTGAATTTTGTCGTGCCAGTAGA | 23 | 63 |
|  | Reverse | GGCAGATGCAAGCGGAGTAA | 20 |  |
| **OsNPR1* | Forward | AAACAAAGGAGCAGCTGTATCACA | 24 | 66 |
|  | Reverse | CTCCGGCAGATACTCATTGCA | 21 |  |
| **OsFMO1* | Forward | CAGTGGAGTGCCCAACATACC | 21 | 65 |
|  | Reverse | CCTGGCCATCAAATGCTTCT | 20 |  |
| **OsActin* | Forward | CAGCCACACTGTCCCCATCTA | 21 | 67 |
|  | Reverse | AGCAAGGTCGAGACGAAGGA | 20 |  |

*Patel 2018, Ashajyothi et al. 2020

**Primers designed using online Platform Primer3Plus <http://www.bioinformatics.nl/cgi-bin/primer3plus/primer3plus.cgi>

**Supplementary TABLE 3|**List of bacterial species used in molecular phylogenetic analysis.

| **Isolates/Strains** | **Gen Bank Accession** | ***Habitat/Source of isolation** | **Closest Match** |
| --- | --- | --- | --- |
| **Gene- rpoC/ 946 bp** |  |  | |
| **OsEnb_ALM_D18** | **ON157423** | **This study** | **OsEn_ALM_D18 (gene- rpoC) with *M. testaceum* StLB037** |
| *M. testaceum* StLB037 | AP012052.1 | Potato leaf endophyte |  |
| *M. chocolatum* SIT 101 | CP015810.1 | Soil |  |
| *M. paraoxydans*DSM 15019 | [LT629770.1](https://www.ncbi.nlm.nih.gov/nucleotide/LT629770.1?report=genbank&log$=nuclalign&blast_rank=3&RID=N963XG9701N) | Clinical samples |  |
| *M. esteraromaticum* B24 | CP043732.1 | Agricultural soil |  |
| *M. hydrocarbonoxydans* Marseille-P2596 | LR882982.1 | Permafrost |  |
| *M. foliorum* M2 | CP041040.1 | Water |  |
| *M. oleivorans*  I46 | CP058316.1 | Soil |  |
| *M. wangchenii* dk512 | CP038266.1 | Tibetan Gazelle |  |

| **Isolates/Strains** | **Gen Bank Accession** | ***Habitat/Source of isolation** | **Closest Match** |
| --- | --- | --- | --- |
| **Gene- tyrS- 759bp** |  |  | |
| **OsEnb_ALM_D18** | **ON157424** | **This study** | **OsEn_ALM_D18 (gene- tyrS) with *M. testaceum* StLB037** |
| *M. testaceum* StLB037 | AP012052.1 | Potato leaf endophyte |  |
| *M. oryzae* MB-10 | CP032550.1 | Rice Field in India |  |
| *M. hominis* PA2F3 | CP054038.1 | Intestinal content |  |
| *M. wangchenii* dk512 | CP038266.1 | Tibetan Gazelle |  |
| *M. sediminis* YLB-01 | CP038256.1 | Deep-sea sediment |  |
| *M. lushaniae* L-031 | CP044232.1 | *Ochotona curzoniae* |  |
| *M. esteraromaticum* B24 | CP043732.1 | Agricultural soil |  |
| *M. caowuchunii* ST-M6 | CP044231.1 | *Ochotona curzoniae* |  |

| **Isolates/Strains** | **Gen Bank Accession** | ***Habitat/Source of isolation** | **Closest Match** |
| --- | --- | --- | --- |
| **Gene- gyrB-771bp** |  |  | |
| **OsEnb_ALM_D18** | **ON157424** | **This study** | **OsEn_ALM_D18 (gene- gyrB) with *M. testaceum* StLB037** |
| *M. testaceum* StLB037 | AP012052.1 | Potato leaf endophyte |  |
| *M. hominis* 01094 | CP061345.1 | Isolated from long term lab culture |  |
| *M. oleivorans* I46 | CP058316.1 | Soil |  |
| *M. aurum* KACC 15219 | CP018762.1 | Corn steep liquor |  |
| *M. paludicola* CC3 | CP018134.1 | Farmland soil |  |
| *M. chocolatum* SIT 101 | CP015810.1 | Soil |  |
| *M. schleiferi* A32-1 | CP064760.1 | Not collected |  |
| *M. lushaniae* L-031 | CP044232.1 | *Ochotona curzoniae* |  |

| **Isolates/Strains** | **Gen Bank Accession** | ***Habitat/Source of isolation** | **Closest Match** |
| --- | --- | --- | --- |
| **Gene- cycS -771bp** |  |  | |
| **OsEnb_ALM_D18** | **ON157424** | **This study** | **OsEn_ALM_D18 (gene- cycS) with *M. testaceum* StLB037** |
| *M. testaceum* StLB037 | AP012052.1 | Potato leaf endophyte |  |
| *M. pygmaeum* DSM 23142 | LT629692.1 | - |  |
| *M. caowuchunii* ST-M6 | CP044231.1 | *Ochotona curzoniae* |  |
| *M. hominis* 01094 | CP061345.1 | Isolated from long term lab culture |  |
| *M. paraoxydans* 70447 | CP064873.1 | *Homo sapiens* |  |
| *M. sediminis* YLB-01 | CP038256.1 | Deep-sea sediment |  |
| *M. paraoxydans* DSM 15019 | LT629770.1 | Clinical samples |  |
| *M. protaetiae* DFW100M-13 | CP035494.1 | *Protaetiabrevitarsis seulensis* larva |  |

| **Isolates/Strains** | **Gen Bank Accession** | ***Habitat/Source of isolation** | **Closest Match** |
| --- | --- | --- | --- |
| **Gene- pyk- 838bp** |  |  | |
| **OsEnb_ALM_D18** | **ON157422** | **This study** | **OsEn_ALM_D18 (gene- pyk) with *M. testaceum* StLB037** |
| *M. testaceum* StLB037 | AP012052.1 | Potato leaf endophyte |  |
| *M. hominis* PA2F3 | CP054038.1 | Intestinal content |  |
| *M. chocolatum* SIT 101 | CP015810.1 | Soil |  |
| *M. oxydans* HG3 | CP031422.1 | Radionuclide-contaminated soil |  |
| *M. lemovicicum* Viu22 | CP031423.1 | Natural uranium-rich soil |  |
| *M. foliorum* 122 | CP019892.1 | Plant |  |
| *M. caowuchunii* ST-M6 | CP044231.1 | *Ochotona curzoniae* (Plateau pika) |  |
| *M. paraoxydans* DSM 15019 | LT629770.1 | Clinical samples |  |

| **Isolates/Strains** | **Gen Bank Accession** | ***Habitat/Source of isolation** | **Closest Match** |
| --- | --- | --- | --- |
| **Gene- metG-784bp** |  |  | |
| **OsEnb_ALM_D18** | **ON157421** | **Rice endophyte: This study** | **OsEn_ALM_D18 (gene- metG) with *M. testaceum* StLB037** |
| *M. testaceum* StLB037 | AP012052.1 | Potato leaf endophyte |  |
| *M. paludicola* CC3 | CP018134.1 | Farmland soil |  |
| *M. hominis* PA2F3 | CP054038.1 | Intestinal content |  |
| *M. paraoxydans* DSM 15019 | LT629770.1 | Clinical samples |  |
| *M. oxydans* VIU2A | CP031338.1 | Radionuclide-contaminated soil |  |
| *M. lemovicicum* Viu22 | CP031423.1 | Natural uranium-rich soil |  |
| *M. wangchenii* dk512 | CP038266.1 | Tibetan Gazelle |  |
| *M. foliorum* M2 | CP041040.1 | Water |  |

| **Isolates/Strains** | **Gen Bank Accession** | ***Habitat/Source of isolation** | **Closest Match** |
| --- | --- | --- | --- |
| **Gene- infB-898bp** |  |  | |
| **OsEnb_ALM_D18** | **ON157420** | **Rice endophyte: This study** | **OsEn_ALM_D18 (gene- infB) with *M. testaceum* StLB037** |
| *M. testaceum* StLB037 | AP012052.1 | Potato leaf endophyte |  |
| *M. paludicola* CC3 | CP018134.1 | farmland soil |  |
| *M. hominis* SJTG1 | CP025299.1 | Wastewater |  |
| *M. oleivorans* I46 | CP058316.1 | Soil |  |
| *M. aurum* KACC 15219 | CP018762.1 | Corn steep liquor |  |
| *M. chocolatum* SIT 101 | CP015810.1 | Soil |  |
| *M. lushaniae* L-031 | CP044232.1 | *Ochotona curzoniae* (Plateau pika) |  |
| *M. wangchenii* dk512 | CP038266.1 | Tibetan Gazelle |  |

| **Isolates/Strains** | **Gen Bank Accession** | ***Habitat/Source of isolation** | **Closest Match** |
| --- | --- | --- | --- |
| **Gene- fumC- 908bp** |  |  | |
| **OsEnb_ALM_D18** | **ON157418** | **Rice endophyte: This study** | **OsEn_ALM_D18 (gene- fumC) with *M. testaceum* StLB037** |
| *M. testaceum* StLB037 | AP012052.1 | Potato leaf endophyte |  |
| *M. aurum* KACC 15219 | CP018762.1 | Corn steep liquor |  |
| *M. ester aromaticum* B24 | CP043732.1 | Clinical samples |  |
| *M. hominis* PA2F3 | CP054038.1 | Intestinal content |  |
| *M. paraoxydans* 70447 | CP064873.1 | *Homo sapiens* |  |
| *M. oxydans* HG3 | CP031422.1 | Radionuclide-contaminated soil |  |
| *M. oleivorans* A9 | CP031421.1 | Radionuclide-contaminated soil |  |
| *M. paludicola* CC3 | CP018134.1 | Farmland soil |  |

*****All the sequences used in the phylogenetic study are from whole-genome sequences.

**Supplementary TABLE 4|** Plant probiotic traits of *Microbacterium testaceum* D18.

| **Probiotic Traits** | **Results** |
| --- | --- |
| Phosphorous Solubilization | + |
| Potassium Solubilization | + |
| Zinc Solubilization | + |
| Siderophore production | + |
| IAA Production | + |
| Ammonia Production | + |
| Cellulase Assay | + |
| Pectinase assay | - |
| Chitinase assay | + |
| Amylase assay | - |
| Proteinase assay | - |

**+ Positive for solubilization/activity; - Negative**

**Supplementary TABLE 5|**Transcriptional response of defense genes in rice upon bacterization by *Microbacterium testaceum.*

| **Defense genes** | **Pusa Basmati 1** | | | | **BPT 5204** | | | |
| --- | --- | --- | --- | --- | --- | --- | --- | --- |
|  | **Seedling bacterization**  **(**CFU mL^-1^) | | **Leaf bacterization**  **(**CFU mL^-1^) | | **Seedling bacterization**  **(**CFU mL^-1^) | | **Leaf bacterization**  **(**CFU mL^-1^) | |
|  | 10^8^ | 10^7^ | 10^8^ | 10^7^ | 10^8^ | 10^7^ | 10^8^ | 10^7^ |
| *OsFMO* | 0.76 | 0.82 | 1.14 | 0.33 | 1.01 | 1.03 | 8.06 | 4.26 |
| *OsPAD4* | 0.66 | 0.51 | 1.95 | 1.77 | 0.79 | 0.70 | 1.49 | 0.68 |
| *OsNPR1* | 1.00 | 0.94 | 1.39 | 2.49 | 0.84 | 0.92 | 2.17 | 0.96 |
| *OsEDS1* | 0.80 | 1.02 | 1.25 | 1.58 | 0.91 | 1.13 | 1.92 | 1.24 |
| *OsCERK* | 1.00 | 1.00 | 3.16 | 2.71 | 1.13 | 1.25 | 0.91 | 0.52 |
| *OsCEBiP* | 0.25 | 0.26 | 2.47 | 0.32 | 0.37 | 0.16 | 1.18 | 0.13 |

*Relative gene expression levels of the transcriptional markers expressed as 2-ΔΔCt values normalized to rice Actin gene expression. Fold change calculations were performed against an untreated control. Data are the mean of three replicates with three plants each. Fold change calculation formula:2^ (Target Ct calibrator-Target Ct sample)*2^(Reference Ct sample-reference Ct calibrator).
